# Polyvinylpyrrolidone Nanofibers Incorporating Mesoporous Bioactive Glass for Bone Tissue Engineering

**DOI:** 10.3390/biomimetics8020206

**Published:** 2023-05-17

**Authors:** Ricardo J. R. Matos, Jorge C. Silva, Paula I. P. Soares, João Paulo Borges

**Affiliations:** 1 i3N/CENIMAT, Department of Materials Science, NOVA School of Science and Technology, NOVA University Lisbon, Campus de Caparica, 2829-516 Caparica, Portugal; rj.matos@campus.fct.unl.pt (R.J.R.M.); jpb@fct.unl.pt (J.P.B.); 2 i3N/CENIMAT, Department of Physics, NOVA School of Science and Technology, NOVA University Lisbon, Campus de Caparica, 2829-516 Caparica, Portugal

**Keywords:** electrospun polyvinylpyrrolidone, polymeric scaffolds, bone regeneration, mesoporous bioactive glass

## Abstract

Composite biomaterials that combine osteoconductive and osteoinductive properties are a promising approach for bone tissue engineering (BTE) since they stimulate osteogenesis while mimicking extracellular matrix (ECM) morphology. In this context, the aim of the present research was to produce polyvinylpyrrolidone (PVP) nanofibers containing mesoporous bioactive glass (MBG) 80S15 nanoparticles. These composite materials were produced by the electrospinning technique. Design of experiments (DOE) was used to estimate the optimal electrospinning parameters to reduce average fiber diameter. The polymeric matrices were thermally crosslinked under different conditions, and the fibers’ morphology was studied using scanning electron microscopy (SEM). Evaluation of the mechanical properties of nanofibrous mats revealed a dependence on thermal crosslinking parameters and on the presence of MBG 80S15 particles inside the polymeric fibers. Degradation tests indicated that the presence of MBG led to a faster degradation of nanofibrous mats and to a higher swelling capacity. The assessment of in vitro bioactivity in simulated body fluid (SBF) was performed using MBG pellets and PVP/MBG (1:1) composites to assess if the bioactive properties of MBG 80S15 were kept when it was incorporated into PVP nanofibers. FTIR and XRD analysis along with SEM–EDS results indicated that a hydroxy-carbonate apatite (HCA) layer formed on the surface of MBG pellets and nanofibrous webs after soaking in SBF over different time periods. In general, the materials revealed no cytotoxic effects on the Saos-2 cell line. The overall results for the materials produced show the potential of the composites to be used in BTE.

## 1. Introduction

Bone transplantation is one of the most common surgical procedures, ranking only behind blood transfusion [1]. Large bone defects result mainly from high-energy traumatic events such as traffic accidents or large bone resection due to different pathologies such as tumors or infections [2]. The body’s innate healing mechanisms frequently cannot spontaneously repair these defects. Bone tissue engineering (BTE) has gained more relevance recently since it can offer promising approaches to bone defects treatment by providing stimulus and osteoconductive templates to facilitate the formation of new bone tissue [3]. Additionally, the biomaterials employed in BTE must possess angiogenic properties, promoting the formation of blood vessels within the constructs. This ensures that the newly regenerated tissue obtains sufficient oxygen and nutrients for its survival [4].

In 1969, BioGlass^®^ 45S5 was first introduced by L. Hench and his collaborators [5]. It is well-established in the literature that BioGlass^®^ 45S5 exhibits non-cytotoxicity and can generate a surface hydroxyapatite (HA) layer that closely resembles the composition of natural bone. This discovery paved the way to a wide range of different bioactive glasses (BGs), thus leading to a wide range of clinical applications in soft and hard tissues (tooth and bone) [6]. Regarding biomedical applications, BG-based materials hold significant importance in dentistry, as BG is capable of creating a chemical bond directly with adjacent tissues and elevating the local pH level to above 5.5; thus, bacteria are deterred from colonizing and thriving at the dental implantation sites, preventing the early stages of caries. Furthermore, the elastic modulus of BG-based materials is comparable to that of the nearby tissues, thus reducing the probability of local bone resorption [7,8,9].

BGs are mostly applied in BTE since they allow the development of scaffolds that possess both osteogenic and angiogenic properties [10]. BioGlass^®^ 45S5 particles have demonstrated interesting results on potential angiogenic effects, considering the increased secretion of vascular endothelial growth factor (VEGF) in vitro and enhancement of vascularization in vivo [11]. Furthermore, Hench and Polak demonstrated that the dissolution products of BGs can exert genetic control over the osteoblast cell cycle, leading to the fast expression of genes that govern osteogenesis [12]. Whether obtained through melting or sol-gel processing, every bioactive glass has the potential to induce bioreactivity or bioactivity by means of its capability to interact with living tissue and form strong bonds to bone. These strong bonds are formed by the precipitation of a hydroxy-carbonate apatite (HCA) layer on the BG surface upon exposure to appropriate physiological fluids or during in vivo applications [13].

Recently, a novel family of mesoporous bioactive glasses (MBGs) exhibiting well-defined mesoporous structures and greater specific surface area has demonstrated remarkable potential for hard tissue engineering applications. The increased interface effects lead to high reactivity, thus improving bioactivity when compared to standard BGs. The synthesis of highly ordered mesoporous materials depends on the use of surfactants as structure-directing agents, which facilitate the formation of channels within the inorganic component. Upon removal of the surfactant, these channels are preserved, resulting in a well-ordered mesoporous structure [14]. In addition, some studies are focusing on improving the properties of BGs by incorporating doping agents instead of manipulating the ratios of different BG components. The doping process aims to improve various aspects of BGs, including their mechanical properties, bioactivity, and biological outcomes, such as promoting angiogenesis, antibacterial activity, bone cell migration, proliferation, and differentiation, or reducing the patient’s inflammatory response. It is possible to incorporate inorganic elements (Ag^+^, Sr^2+^, Cu^2+^, Zn^2+^, Mg^2+^, etc.) or organic compounds such as VEGF, bone morphogenetic proteins (BMPs), dexamethasone (DEX), gentamicin, etc. [15]. For example, Bellucci et al. studied the effect of replacing 10 mol% of CaO with MgO or SrO from a silicate BG. The researchers reported that the doped BGs, especially the Mg-doped, further improved the bioactivity [16]. Tang et al. demonstrated that BGs loaded with rhBMP-2 promoted excellent cell attachment, ingrowth, and osteogenesis in vitro and supported complete bone regeneration in vivo using a rabbit radius critical size defect model [17]. Lim et al. produced electrospun nanofiber matrices that incorporated BG nanoparticles loaded with DEX. The research group reported that these scaffolds promoted the odontogenesis of human dental pulp cells (HDPCs) [18].

BGs and MBGs can be produced to feature different morphologies (e.g., powder, granules, monoliths, fiber, coatings, etc.) [19]. In this application area, it is important to take into account both micro-sized and nanoscale powder particles, which can be used in the production of composite material scaffolds, such as the combination of electrospun biodegradable polymers and MBGs, which is the purpose of this work. Several studies have been carried out in order to combine polymers with inorganic phases such as BGs or HA. In this context, Petretta et al. have reported the production of composite scaffolds based on polycaprolactone (PCL) and Mg-containing bioactive glasses [20]. In general, the main benefit of using these composite materials is that they retain the bioactivity of the inorganic bioactive filler while preserving the mechanical properties of the polymer, such as flexibility and the ability to deform under loads. Moreover, the incorporation of MBG powder nanoparticles in a polymeric mat mimics the natural bone structure more accurately as it consists of nanoscale hydroxyapatite crystallites together with the polymeric phase of collagen type I. This natural combination is responsible for the mechanical properties of bone [13]. From this perspective, the size of the BG or MBG particles incorporated into the polymeric matrices also plays an important role in enhancing bioactivity. Research studies suggest that replicating the nanoscale characteristics of bone can enhance the attachment and growth of bone-forming cells [21].

Polyvinylpyrrolidone (PVP) is a synthetic polymer that exhibits high solubility in water as well as numerous organic solvents. PVP has found extensive application in the production of hydrogels, wound dressings, nanofibers/scaffolds, and drug and gene delivery systems. During World War II, PVP gained significant recognition as a blood plasma substitute [22].

The ability of the electrospinning technique to produce nanofibrous scaffolds that mimic ECM while incorporating other materials in particulate form makes it the ideal technique to produce composite scaffolds for bone tissue engineering [23,24]. In this paper, we have successfully prepared MBG 80S15 by using Pluronic F127 as a structure-directing agent through an evaporation-induced self-assembly (EISA) process. Nanosized MBG 80S15 powder was further incorporated into electrospun PVP nanofibers, and its properties were studied to assess the potential of such composites for use in BTE applications. Furthermore, the coupling of PVP with MBG nanoparticles produces composite 2D materials where it is possible to control the degradation and bioactivity rates to fit the natural processes of bone repair.

## 2. Materials and Methods

Analytical-grade chemical reagents were utilized in this research without the need for additional purification.

### 2.1. Synthesis of Mesoporous Bioactive Glass MBG 80S15

Mesoporous Bioactive Glass MBG 80S15 is a ternary bioactive glass (SiO_2_–CaO–P_2_O_5_) which is produced by a sol-gel technique adapted from a previously described method by Yan et al. [25]. MBG was synthesized by using the nonionic block copolymer Pluronic F127 (EO_106_PO_70_EO_106_) (Sigma-Aldrich, Lisbon, Portugal) as structure-directing agent (EO is poly(ethylene oxide), and PO is poly(propylene oxide)).

In a typical synthesis of MBG 80S15, in order to achieve a molar ratio of Si/Ca/P = 80:15:5, tetraethyl orthosilicate (Sigma-Aldrich) (TEOS, 6.7 g), calcium nitrate-4-hydrate (PanReac AppliChem, Darmstadt, Germany) Ca(NO_3_)_2_·4H_2_O (1.4 g), triethyl phosphate (Sigma-Aldrich) (TEP, 0.73 g), and 0.5 M HCl (1.0 g) were dissolved in absolute ethanol (Sigma-Aldrich) (60 g). TEOS, TEP and calcium nitrate-4-hydrate were used as precursors of SiO_2_, P_2_O_5_ and CaO, respectively.

The mixture was allowed to react for 30 min at room temperature under magnetic stirring at 250 rpm (2Mag Mix 15 Eco Stirrer, Germany) for the acid hydrolysis of TEOS. Then, 1.82 g of Pluronic F127 was added to the mixture and the resultant solution was also stirred at room temperature for 60 h to undergo an evaporation-induced self-assembly (EISA) process. The gel obtained was heated at 60 °C (Memmert Incubator Modell 100–800, Schwabach, Germany) under atmospheric pressure for 24 h to completely remove the alcohol. Subsequently, the dried gel was calcined in a muffle furnace (Nabertherm GmbH HTCT 01/16, Lilienthal, Germany) at 700 °C for 5 h (ramp of 2 °C·min^−1^) to obtain the final MBG 80S15 products. The resulting powder was ground in a planetary ball mill (Planetary Mono Mill Pulverisette 6—Fritsch, Idar-Oberstein, Germany) for 6 h at 400 rpm using zirconia balls with a 2 mm diameter. The final average grain size obtained, according to SEM images using ImageJ software, (version 1.52n, National Institutes of Health, Bethesda, MD, USA), was about 174 ± 119 nm (100 measurements).

### 2.2. Optimization of Electrospinning Process by DOE

A solution of 18% *w*/*v* PVP ($M_{w}\;\sim \;1,300,000\;g/\mathrm{mol})\;$(Sigma-Aldrich) in ethanol (Sigma-Aldrich) was prepared for electrospinning, which was carried out at a temperature of 20 °C with a humidity level of approximately 40%.

In this study, the environmental conditions and the solution parameters were kept constant. Four operating variables (flow rate, tip-collector distance, applied voltage, and needle gauge) were studied by applying a custom Design of Experiments and response surface methodology (RSM) as a statistical method. The custom DOE was performed using JMP software, version 13.0 (S.A.S. Institute Inc., Cary, NC, USA) to investigate the effect of above-mentioned electrospinning process parameters and the interactions between these variables on the average electrospun PVP fiber diameter.

The range of each variable was chosen considering the set of values that allow the PVP electrospinning process based on the literature and previous experiments (Table 1). Within the range defined for each variable, the software selected a set of experiments consisting of a minimum of 27 runs. To determine how to optimize the parameters, several outputs were measured: the samples were analyzed by SEM to observe the overall structure of the collected mats and the average fiber diameter was calculated using ImageJ Software by selecting at least 25 random fibers [26]. Using the RSM, the overall desirable response (the set of variables that minimizes the average fiber diameter) was obtained from the software’s calculations within the pre-selected range of each variable. The software also estimated the resulting average PVP fiber diameter for that set of variables, which was about 652 ± 179 nm.

**Figure 1:**
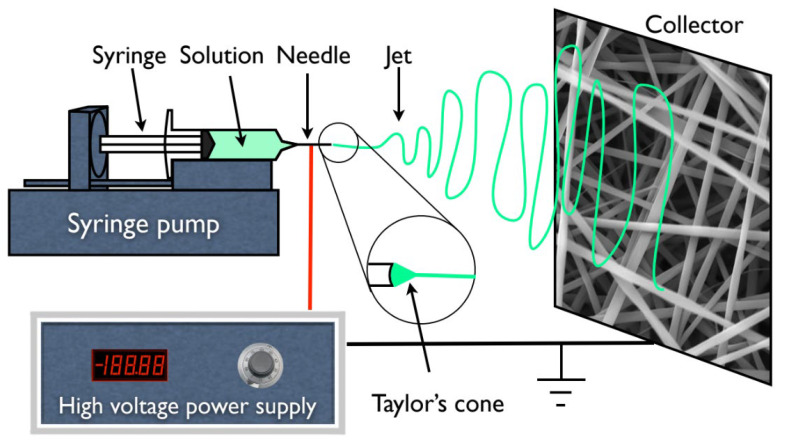
Schematic representation of an electrospinning apparatus in horizontal setup with a static plate collector.

In addition, for all the experiments and subsequent membrane production, a 1 mL syringe was loaded with the polymer solution which was dispensed using a syringe pump (KDS100, KD Scientific, Holliston, MA, USA) at different rates from 0.1 to 0.4 mL·h^−1^. A high voltage supply (Glassman High Voltage INC EL 30kV, High Bridge, NJ, USA) was connected to the metallic needle (ITEC Iberiana Technical, Braga, Portugal), and a sheet of aluminum foil was used as collector and placed in front of the needle tip (schematically represented in Figure 1) at distances ranging from 15 to 20 cm to collect the fibers.

#### 2.2.1. PVP/MBG Composite Production

To obtain the solution for electrospinning, the MBG powder was first combined with ethanol and subsequently sonicated for 15 min at 320 W for further dispersion. PVP was weighed and dissolved in the previously dispersed solution containing the solvent to obtain a final polymer concentration of 18 *w*/*v* %. The final blend was magnetically stirred for at least 30 min, resulting in a well-dispersed MBG powder in the polymeric solution. Different percentages of the MBG powder were added to ethanol for further dispersion: 10; 28; 56; 83; and 100% by weight with respect to PVP content. The quantity of MBG powder utilized in this study must be significant to promote bioactivity and regulate the degradation rate of PVP to some extent [27]. In this context, the composites containing 100% MBG by weight with respect to PVP are of major interest. This composite will be denominated PVP/MBG (1:1) as the mass content of MBG and PVP is the same.

Electrospinning was carried out with the same processing parameters estimated from the DOE to plain PVP, except for the needle gauge. The composites were electrospun with a G23 needle (ITEC Iberiana Technical, Braga, Portugal) to prevent clogging. The membranes produced were kept in a desiccator until the crosslinking treatment due to the water solubility of PVP.

#### 2.2.2. Thermal Crosslinking

The water solubility of PVP restricts the use of electrospun PVP-based nanofibers without any further treatment. Nonetheless, the solubility of PVP can be reduced by heating it in air between 150 and 200 °C [28]. The as-spun PVP and PVP/MBG composites were crosslinked in an incubator (Memmert Incubator Modell 100–800, Schwabach, Germany) at different temperatures within the above-mentioned range. Final temperatures of 150, 165, and 180 °C were studied for different times: 3, 4, 5, 8, 12, and 24 h. To investigate the effect of thermal crosslinking, the membranes were immersed for 24 h in water, dried at 60 °C and analyzed by SEM.

### 2.3. Characterization

The images captured using transmission electron microscopy (TEM) were acquired using a Hitachi H-8100II (Tokyo, Japan) instrument equipped with thermo-ionic emission LaB6, offering a resolution of 2.7 Å. The analysis of the membranes was conducted on the thinnest membrane that could be technically extracted (around 15–20 min of deposition).

Powder X-ray diffraction (XRD) was employed to confirm the crystalline phases present in the samples. X-ray diffraction patterns were obtained using an X’Pert PRO PANAlytical X-ray diffractometer (Malvern, UK). The measurements were taken over a range of 2θ values from 15° to 80°, using Cu-Kα radiation (λ = 1.54060 Å) with a step size of 0.033.

The Nicolet 6700-Thermo Electron Corporation Attenuated Total Reflectance-Fourier Transform Infrared (ATR-FTIR) spectrometer (Watertown, WI, USA) was employed to obtain the FTIR spectra of the samples. The measurements were taken in the 480–4000 cm^−1^ range with a resolution of 2 cm^−1^.

Carl Zeiss Auriga (Oberkochen, Germany) scanning electron microscopy (SEM) equipment was used to analyze the fiber morphology. The samples were previously coated with a thin layer of either iridium–palladium or carbon, depending on which elements were being studied by complementary EDS analysis.

N_2_ adsorption–desorption isotherms were measured with a Gas Porosimeter Micromeritics (ASAP 2010, Norcross, GA, USA) at −196 °C. Prior to the measurements, the samples underwent a 12 h outgassing process at 300 °C in vacuum. The specific surface area was calculated using the Barrett–Emmett–Teller (BET) method. The pore volume and pore size distribution were determined using the Barrett–Joyner–Halanda (BJH) method based on the adsorption branches of the isotherms. The total pore volume was estimated from the adsorbed amount at a maximum relative pressure.

Thermo-gravimetric analysis (TGA) curves were recorded with the Simultaneous Thermal Analyser (STA 449 F3 Jupiter Netzsch (Selb, Germany)) under an inert nitrogen atmosphere at a heating rate of 10 °C/min from room temperature to 900 °C. Differential TGA (DTGA) was obtained by differentiating the respective TGA curve.

### 2.4. Swelling and Degradation Assays

The success of thermal crosslinking was studied by testing the dissolution of crosslinked mats immersed in phosphate buffer solution (PBS, pH 7.4) at 37 °C at different time points: 1, 3, 7, 13, 17, 21 and 28 days. At these time points, the degradation of PVP and PVP/MBG composites fibers in physiological solutions was assessed by measuring the membranes’ weight loss. The samples were removed from PBS, gently rinsed with Millipore water to remove saline and dried at 37 °C until constant mass was reached The percentage of weight loss ($W_{L}\;\%$) was computed as $W_{L}\;\%=100\times \frac{\left(W_{0}-W_{r}\right)}{W_{0}}\;$, where $W_{0}$ and $W_{r}$ are the initial and the residual weight of the sample, respectively.

The swelling ratio is a measure of the liquid-absorbing capacity of a matrix. The dry weight of each sample was measured ($W_{d}$) before soaking in PBS (pH 7.4). The mass of each sample was planned to be at least 10 mg, ensuring the membrane stability over the experiment. At pre-determined intervals of time (5, 15, and 30 min; 1 and 5 h; 1, 3, and 7 days) the specimens were removed from the PBS. The excess liquid on each matrix was removed, and the wet matrices were weighed ($W_{w}$). The swelling ratio (Q) was calculated as: $Q=\frac{W_{w}-W_{d}}{Wd}$.

In both degradation and swelling experiments, all samples were cut with dimensions of 20 mm × 20 mm and placed in a plastic container (Falcon tube, 50 mL) filled with PBS (pH 7.4). The experiments were performed in an incubator with an orbital shaker (Comecta—Heated Incubator with Shaker S-100D, Barcelona, Spain) at 37 °C. An average of at least three samples was taken for each time point. The samples were weighed with an accuracy of 0.1 mg on an analytical balance (Sartorius Quintix Model 224-1S, Goettingen, Germany).

### 2.5. Mechanical Response—Tensile Tests

The tensile tests were carried out using a Rheometric Scientific uniaxial tensile testing machine equipped with a 20 N load cell and controlled by Minimat software (Minimat Control Software Version 1.60 February 1994 (c) PL Thermal Science 1984-94 Rheometric Scientific Ltd., New Castle, DE, USA). All experiments were conducted at room temperature and approximately 50% humidity, with a velocity of 1 mm·min^−1^. At least 10 samples of each membrane type were tested, and each sample was cut into a rectangular shape measuring 10 mm in width and 20 mm in length. The thickness of the samples was measured using a digital micrometer (Mitutoyo 0–25 mm, Aurora, IL, USA). The stress–strain curves were used to determine the ultimate tensile strength, Young’s modulus and yield strength.

### 2.6. Cytotoxicity Assays

The cytotoxicity of the membranes was evaluated using the extract method and the Saos-2 cell line (American Type Culture Collection, HTB-85), following the standard ISO-10993 for the biological evaluation of medical devices, specifically Part 5: tests for in vitro cytotoxicity. To produce the extract, different samples were cut to have an identical mass of 30 mg and pre-sterilized at 120 °C for 2 h. Each sample was placed in 1.5 mL (resulting in an extract concentration of 20 mg·mL^−1^) of McCoy 5A culture medium supplemented with 2.2 g/L sodium bicarbonate (both from Sigma Aldrich, Portugal), penicillin (100 U/mL) and streptomycin (100 µg/mL) (Gibco Life Technologies, Billings, MT, USA) and 10% FBS (Fetal Bovine Serum, S. America origin, Biowest, Nuaillé, France) at 37 °C for 48 h.

Cells were seeded at a density of 30,000 cell·cm^−2^ in 96-well plates and grown in fully supplemented McCoy followed by incubation at 37 °C in 5% CO_2_ for 24 h. The subsequent step was to exchange the medium for the extract to assess whether the cells would survive the next 48 h. Five concentrations of the extract were used: 20, 10, 5, 2.5 and 1.25 mg·mL^−1^ (two-fold serial dilution). Four replicates were used for each concentration. After this period, the culture medium was removed, and a solution of resazurin (Alfa Aesar, MA, USA) containing 10% 0.2 mg·mL^−1^ resazurin solution in PBS and 90% complete culture medium was added to each well. After a 2-h incubation period, the absorbance was measured at 570 nm and 600 nm using a multi-well plate reader (BioTek ELX800 UV, Winooski, VT, USA). The negative control cells were cultured in complete medium while positive control cells were exposed to 10% DMSO to induce cell death. Cell viability was determined as a percentage of the negative control, calculated by [% cell viability = treated cells/control cells × 100].

### 2.7. Bioactivity Assay

The bioactivity of PVP/MBG composites, related to the surface deposition of hydroxy-carbonate apatite (HCA) layers, was evaluated by immersing the samples in simulated body fluid (SBF) solution. The SBF had a composition and ionic concentration similar to those of human body plasma, and it was prepared by dissolving respective amounts of the reagent chemicals (NaCl (Sigma-Aldrich), NaHCO_3_ (Sigma-Aldrich), KCl (Sigma-Aldrich), MgCl_2_·6H_2_O (Sigma-Aldrich), HCl (Honeywell Fluka, Lisbon, Portugal), CaCl_2_ (Carl Roth, Karlsruhe, Germany), Na_2_SO_4_ (Sigma-Aldrich), K_2_HPO_4_·3H_2_O (VWR, Lisbon, Portugal), and (CH_2_OH)_3_(CNH)_2_ (Apollo Scientific, Cheshire, UK) as the buffering agent) into deionized water according to the procedure described by Kokubo et al. [29,30]. The prepared SBF was buffered at pH 7.4 with tris (hydroxymethyl aminomethane) and 1 M hydrochloric acid at 37 °C. The polymeric matrices were cut into samples of 15 × 15 mm and subsequently immersed in 50 mL SBF in a falcon tube for different time periods (1, 3, 5 and 10 days). This experiment followed international standards ISO/FDIS 23317 [31]; the volume of the SBF solution $\left(V_{SBF}\right)$ should have been at least the volume (mL) determined according to the following formula: $V_{SBF}=\frac{S_{A}}{10}$, where $S_{A}$ is the surface area of the samples (mm^2^). During the experiment, the samples were kept at 37 °C in an incubator with orbital shaker (54 rpm), and the SBF solution was refreshed every two days. After the soaking periods, the samples were removed from SBF, rinsed with Millipore water and dried at room temperature. The bioactivity assay was performed for both the PVP/MBG (1:1) composites and MBG powder. For the latter, it was necessary to produce pellets by dry pressing. Each pellet was produced from 300 mg of powder in a hardened steel mold with an inner diameter of 13 mm. The powder was pressed uniaxially at a load of 5 tonnes for 5 min at room temperature.

Formation of a bone-like apatite layer on the surface of the nanocomposite mats was determined by X-ray diffraction (XRD), Fourier transform infrared spectroscopy (FTIR) analysis and scanning electron microscopy coupled with energy dispersive spectroscopy (SEM–EDS).

## 3. Results

### 3.1. Mesoporous Bioactive Glass MBG 80S15 

MBG synthesis was performed by the sol-gel method using Pluronic F127 as a structure-directing agent. During the synthesis process, the evaporation of organic solvent progressively increases block copolymer concentration. When the concentration of Pluronic F127 reaches critical micelle concentration, micellar aggregates form through the self-assembly process of silica-surfactant composite micelles, which organize into liquid-crystalline mesophases. Calcination allows the removal of the organic surfactant template and retention of the inorganic framework to obtain highly ordered mesostructured bioactive glass materials [14,32]. 

**Figure 2:**
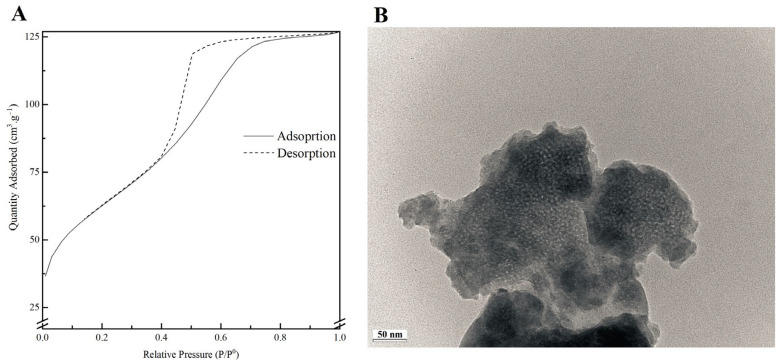
Nitrogen adsorption-desorption isotherms (**A**) and TEM image (**B**) of MBG 80S15 powder.

Figure 2A shows the nitrogen adsorption–desorption isotherms of the MBG 80S15 calcined powder. The isotherms obtained exhibit a type IV pattern, which is consistent with a mesoporous structure. Additionally, at a relative pressure of approximately 0.40, the sample displays type H1 hysteresis loops, which are indicative of cylindrical pores, in accordance with the p6mm mesostructure. The BET surface area of the MBG calculated from the linear part of the BET plot reached 232 m^2^·g^−1^. The pore size distribution curves were obtained by applying the BJH model to the adsorption and desorption branches. The MBG presents a relatively narrow pore size distribution, and the peak pore size was around 5.9 nm and 6.0 nm for adsorption and desorption branches, respectively. This proximity between pore size results can be an indicator of a perfectly cylindrical channel. The pore volume was also calculated using the BJH model and the result was about 0.25 cm^3^·g^−1^ for both the adsorption and desorption branches.

A TEM image of MBG 80S15 is shown in Figure 2B. Brighter spot areas, which can be an indicator of the expected mesoporous structure, can be observed. However, the cylindrically ordered structure of the mesoporous BG is not evident. Analysis of the sample structure by TEM is hindered by powder aggregation, which increases the thickness and the opacity of the samples. Moreover, the TEM equipment resolution also limits the proper visualization of the MBG mesoporosity.

**Figure 3:**
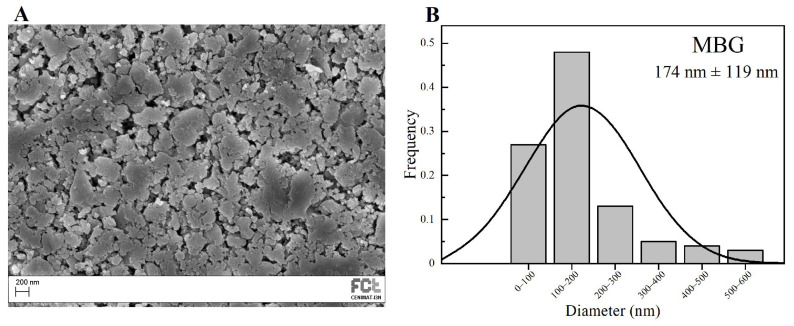
SEM image of MBG 80S15 pellet (**A**) and the respective particle size distribution graph after 6 h of ball-milling (**B**).

SEM image of the MBG 80S15 obtained after 6 h of planetary ball-milling is presented in Figure 3A. It can be observed that the size of MBG particles is in the nanoscale range, as shown in the respective particle size distribution graph Figure 3B. The average diameter of MBG particles is around 174 ± 119 nm. It is worth pointing out the reduced dimensions of these particles, considering they become easier to disperse in an electrospinning solution compared to micro-sized MBG particles.

**Figure 4:**
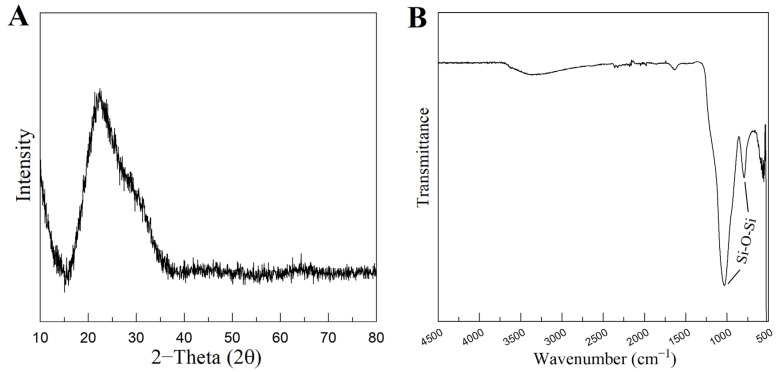
XRD pattern (**A**) and FTIR spectra (**B**) of MBG 80S15 powder calcined at 700 °C for 5 h.

Figure 4A represents the XRD pattern of the produced MBG 80S15. It can be observed that there are no diffraction peaks appearing on the pattern of the sample, except for a broad reflection at 2θ = 15–35° (characteristic of silicate glasses) which suggests that the MBG 80S15 produced exists in an amorphous state. The temperature assigned to the occurrence of crystallization is about 810 °C [32]. It is important to note that MBG was calcinated at 700 °C to ensure that the mesoporous structure retained its stability. When MBGs are calcined at temperatures higher than 810 °C, the inorganic wall becomes crystalline and the mesostructure collapses.

Figure 4B shows the FTIR spectrum of the produced MBG 80S15 powder. It is shown that the sample exhibits the characteristic absorption bands of a silicate glass. The spectrum shows the Si-O-Si asymmetrical stretching vibration at 1040 cm^−1^ and Si-O-Si symmetrical stretching vibration at 806 cm^−1^. The band associated with Si-O bending (rocking) vibration at 470 cm^−1^ was not detected due to the equipment resolution (the measuring process starts at 500 cm^−1^) [33,34].

### 3.2. PVP/MBG 80S15 Composites

**Figure 5:**
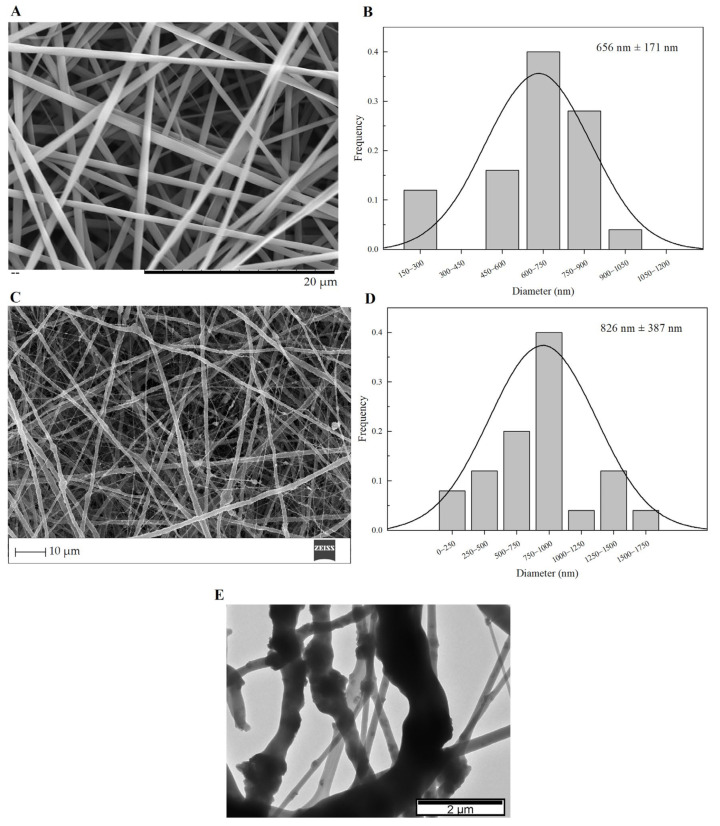
SEM images and the respective fiber diameter distribution graph of plain PVP membrane (**A**,**B**), and PVP/MBG (1:1) composite (**C**,**D**). TEM image of PVP/MBG (1:1) composite (**E**).

Custom Design of Experiments and RSM are statistical methods that allow the definition of a set of electrospinning processing parameters that minimize average PVP fiber diameter as described above (Section 2.2). JMP software (version 13.0) estimated an average resulting PVP fiber diameter of about 652 ± 179 nm for this set of parameters: flow rate of 0.10 mL·h^−1^; applied voltage of 18.6 kV; tip-collector distance of 17.3 cm; needle gauge G27. The SEM image and respective average size distribution are shown in Figure 5A,B. SEM micrographs revealed that the electrospun PVP nanofiber mats were composed of randomly oriented, uniform and bead-free nanofibers, with an average diameter size of 656 ± 171 nm, which is quite close to the estimated value from DOE.

The PVP/MBG composites were electrospun in the same conditions as plain PVP, except for the needle gauge (G23) to avoid clogging. The SEM image of PVP/MBG (1:1) composite and the respective fiber size distribution are represented in Figure 5C,D. It can be observed that the incorporation of MBG particles into the PVP fibers changes the fiber’s surface. The plain PVP fibers have a smooth surface, which becomes rough and irregular when the MBG particles are incorporated into the fibers, as expected due to the particles’ aggregates as shown in the TEM image (Figure 5E). Consequently, the average fiber diameter and standard deviation of PVP/MBG (1:1) composite fibers increase to 826 nm and 387 nm, respectively. However, composite fibers exhibited a relatively homogeneous distribution of embedded MBG 80S15 particles. 

**Figure 6:**
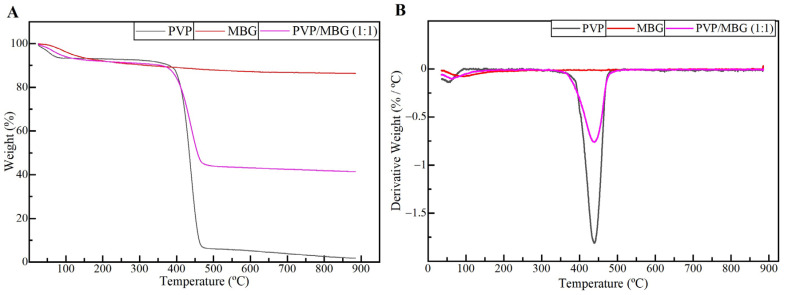
TGA (**A**) and DTGA (**B**) curves of plain PVP (black), PVP/MBG (1:1) composite (purple), and MBG 80S15 calcined at 700 °C (red).

TGA analysis was performed on plain PVP, MBG 80S15, and composite electrospun mats PVP/MBG (1:1). The TGA and DTGA curves obtained are reported in Figure 6A,B. All samples show a slight initial mass loss (around 9%) at 125 °C, attributed to the loss of the physically adsorbed water. The thermogram curve for MBG 80S15 previously sintered at 700 °C does not show another considerable mass loss, thus presenting a residual mass of 86.4% at 900 °C. Meanwhile, the thermogram curve of the plain PVP membrane exhibited a steep loss in weight between 400 and 480 °C, and there was no significant residual mass left after heating to 900 °C, indicating complete degradation of the polymer. In contrast, for PVP/MBG (1:1) nanocomposite fiber mats, a weight loss of 58.6% was observed within the tested temperature range. This weight loss is associated with water removal and PVP degradation. The residual mass of 41.4% corroborated the presence of MBG in the nanocomposite mats. The residual mass is consistent with the theoretical amount of MBG content in the composite, considering the initial amount introduced in the electrospinning solution. These results, along with the SEM and TEM analyses, provided confirmation that composite structures were effectively prepared in the present study.

### 3.3. Swelling and Degradation Assays

#### 3.3.1. Thermal Crosslinking

**Figure 7:**
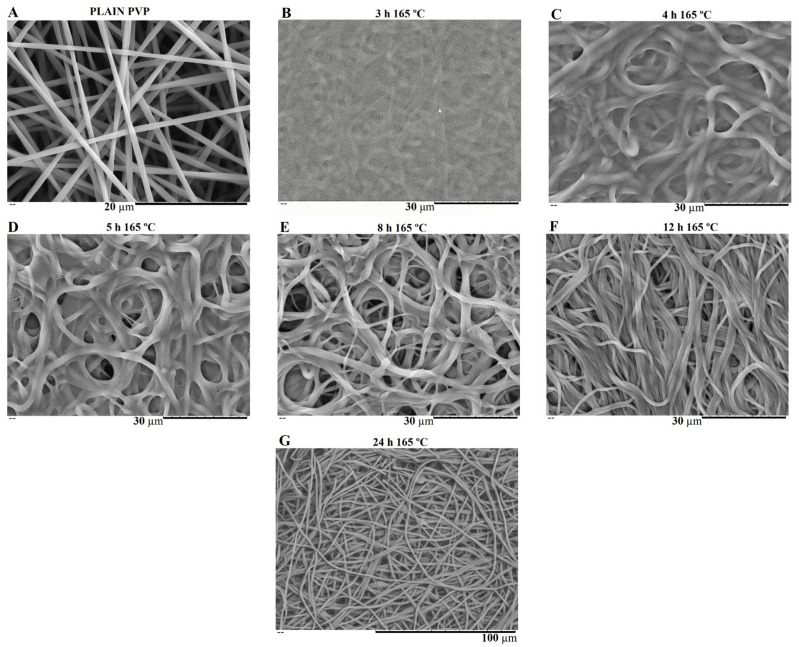
SEM image of electrospun plain PVP before soaking into PBS (**A**), and SEM images of plain PVP thermally crosslinked at 165 °C for 3 h (**B**), 4 h (**C**), 5 h (**D**), 8 h (**E**), 12 h (**F**) and 24 h (**G**) after soaking in PBS for 1 day.

**Figure 8:**
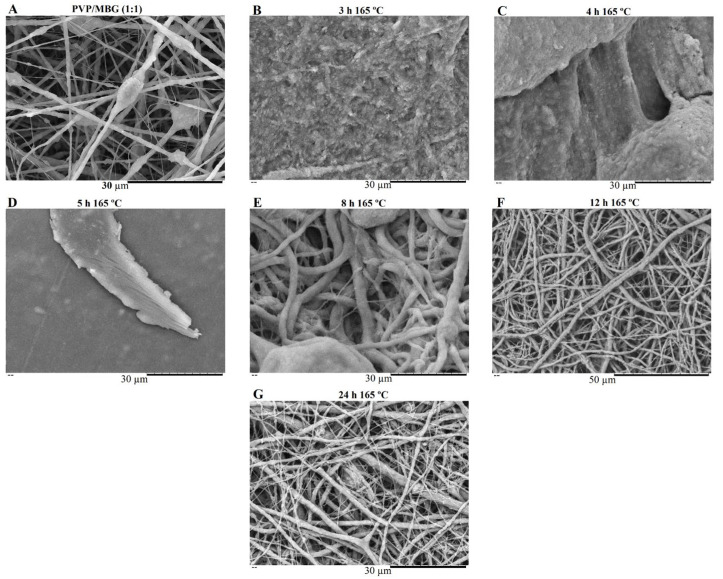
SEM image of electrospun PVP/MBG (1:1) composite before soaking in PBS (**A**) and SEM images of PVP/MBG (1:1) composites thermally crosslinked at 165 °C for 3 h (**B**), 4 h (**C**), 5 h (**D**), 8 h (**E**), 12 h (**F**) and 24 h (**G**) after soaking in PBS for 1 day.

PVP is water soluble, thus the as-spun nanofibers need to be crosslinked to make them water resistant and suitable for bone tissue engineering (BTE). Thermal treatment was found to be useful as a crosslinking agent of polymer chain segments, as it is easy to apply. Several crosslinking conditions were tested depending on the temperature applied (range: 150–200 °C) and time (range: 3–24 h) [28,35]. However, at a temperature of 165 °C, the membranes showed the most promising results in terms of reducing PVP solubility by keeping the overall nanofiber structure without burning. This temperature was kept constant, and different crosslinking times were tested as shown in Figure 7 and Figure 8. 

SEM images of Figure 7 show the electrospun plain PVP before and after soaking in PBS for 24 h, depending on previous crosslinking time. Figure 8 shows a similar study for PVP/MBG (1:1) composites, to compare how the MBG particles incorporation affects the thermal crosslinking. Both pure PVP and PVP/MBG (1:1) thermally crosslinked for 3 h appear to lose their fibrous structure and form a kind of rough film without porosity. However, several differences were noticed: the fibrous structure of the plain PVP membranes crosslinked for 4 and 5 h was not completely lost but appears as if the adjacent fibers have fused together and are significantly reduced in porosity, whereas the composites crosslinked for 4 and 5 h completely lose their fiber structure. In the case of the membranes crosslinked for 8 h, the fibrous structure of the membranes is still observed in both plain PVP and PVP/MBG (1:1), but the thickening of the fibers was clearly observed. In general, as crosslinking time increases, most of the morphological characteristics were observed to remain unchanged. The results also show that MBG nanoparticles’ incorporation into the PVP fibers also reduces the degree of composite crosslinking for the same crosslinking time and temperature when compared to pure PVP. The MBG incorporation yielded thicker and more heterogeneous fibers; thus, the effect of the temperature on PVP crosslinking can be slightly affected due to the filtering effect [36].

#### 3.3.2. Degradation Assay

**Figure 9:**
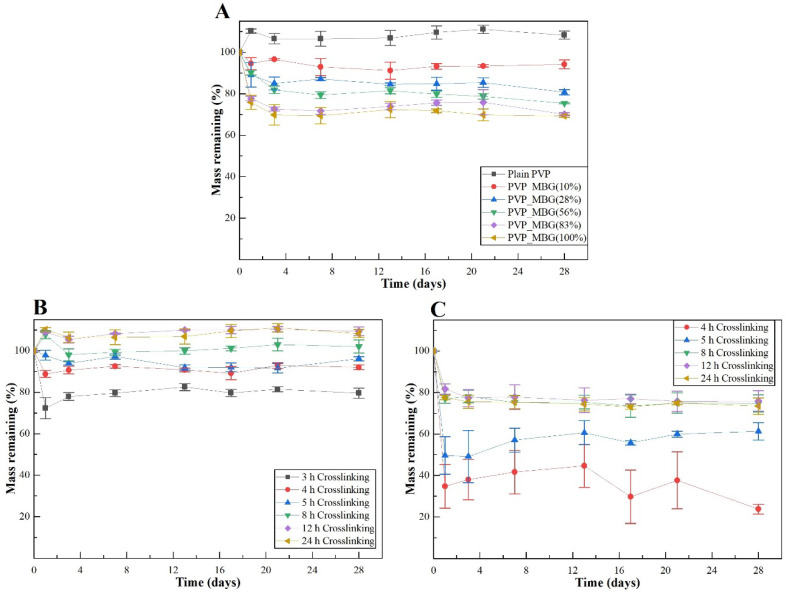
Degradation assay for PVP/MBG composites (thermally crosslinked at 165 °C for 24 h) with different MBG 80S15 content: 0, 10, 28, 56, 83 and 100% of PVP mass (**A**); degradation assay for electrospun PVP membranes (**B**) and PVP/MBG (1:1) composites (**C**) thermally crosslinked at 165 °C for different times: 3, 4, 5, 8, 12 and 24 h.

The rate at which a scaffold degrades is an essential parameter in bone tissue engineering, as it should match the rate of ECM neogenesis. The duration necessary for bone recovery varies based on the fracture’s shape and location, the condition of the adjacent soft tissues and individual factors such as species, age, general health and concurrent illnesses or injuries. In general, bone healing time ranges from 6 weeks to 6 months [37,38,39]. Therefore, in vitro biodegradation was studied by measuring weight loss of fiber mats in PBS at 37 °C for 4 weeks. Figure 9A represents the degradation ratio of PVP/MBG composites thermally crosslinked at 165 °C for 24 h with different amounts of MBG incorporated into the PVP fibers, ranging from 10 to 100% of the polymer’s mass. Pure PVP (also described as plain PVP) was used as a control and revealed no mass loss during the assay, while the composite with a weight percentage of MBG equal to 100% of PVP mass (also mentioned as PVP/MBG (1:1)) revealed a mass loss around 30% after 28 days. Moreover, the data show that scaffold mass loss increases with MBG content for the same soaking time in PBS. This indicated that the inclusion of MBG particles accelerated the degradation rate of the fibrous mats. It was explained as being due to the high affinity of the MBG 80S15 with water, which increased the ability of the composites to absorb and retain water from the medium during the incubation periods and, consequently, raised the hydrolytic degradation of PVP fibers [40]. The MBG dissolution over time also explains the increasing mass loss of composites with higher MBG contents.

Figure 9B,C represent the degradation assay for electrospun plain PVP membranes and PVP/MBG (1:1) composites, respectively. The samples were thermally crosslinked at 165°C for different times: 3, 4, 5, 8, 12 and 24 h. Generally, polymer degradation kinetics is affected by chemical and structural characteristics. In general, both graphics show that the degradation ratio decreases as the crosslinking times increase, except for the PVP/MBG (1:1) composites crosslinked for 8, 12 and 24 h, which presented similar degradation ratios over time. This behavior was expected because thermal crosslinking can enhance the thermal and chemical stabilities of PVP to form a stable network structure [41]. However, under the same thermal crosslinking conditions, the incorporation of MBG accentuated the degradation ratio compared to pure PVP. Furthermore, membranes with a low degree of crosslinking quickly led to the high exposure of MBG particles to the medium. Consequently, the particles could be separated from PVP fibers, thus increasing the degradation ratio even further. The degradation ratio data of PVP/MBG (1:1) thermally crosslinked for 3 h is not shown in the graph because the samples partially lose their physical integrity. It is worth pointing out that all samples studied showed a fast degradation ratio during the first 2 h, then degradation was very slow or reached a plateau. 

In general, the degradation ratios studied are in agreement with the overall structural changes of the samples after 24 h of immersion in PBS (Figure 7 and Figure 8).

#### 3.3.3. Swelling Assay

**Figure 10:**
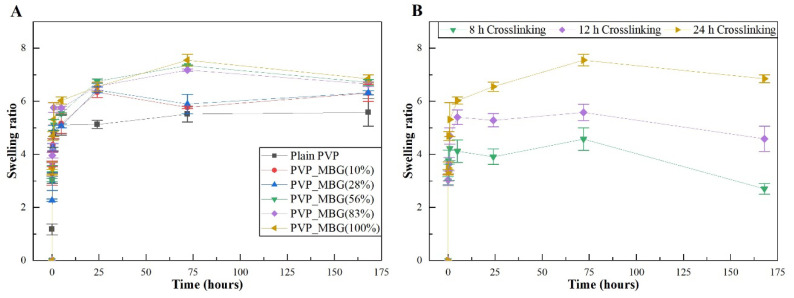
Swelling assay for PVP/MBG composites (thermally crosslinked at 165 °C for 24 h) with different MBG 80S15 content: 0, 10, 28, 56, 83, and 100% of PVP mass (**A**); Swelling assay for PVP/MBG (1:1) composites crosslinked for different times: 8, 12, and 24 h (**B**).

Figure 10A,B show the swelling assay for different PVP/MBG composites depending on MBG content and thermal crosslinking duration, respectively. For each of the membranes studied, the water absorption continues to increase until equilibrium is reached (approximately 24 h of incubation). Plain PVP was used as a control and its swelling ratio was calculated at around 500%, whereas the data show as the amount of incorporated MBG increases, the swelling ratio also increases. The PVP/MBG (1:1) composite had the highest swelling ratio around 750–800%. The hydrophilic character of PVP, the high surface area of the matrices and the presence of MBG nanoparticles inside the fibers are factors that positively affect the swelling capability of the scaffolds since it is dependent on the degree of water uptake by pure polymer or composites. On the other hand, the PVP/MBG (1:1) composites crosslinked for 8, 12, and 24 h showed different swelling capacities (Figure 10B). As discussed above, Figure 8E–G illustrate the respective composites after 24 h of immersion in PBS. It can be noticed that PVP fibers reduce the structural changes after being immersed in PBS as the crosslinking time increases, thus the water uptake from the medium and the capacity to retain such water reaches higher levels. Another study also reported the degree of swelling of polyester elastomers is an important parameter in characterizing their crosslinking degree [42]. Scaffolds with swelling capacity are highly desirable, particularly for drug delivery purposes where the drug can be trapped in the polymeric composite material and then released in a controlled manner.

### 3.4. Mechanical Response—Tensile Tests

**Figure 11:**
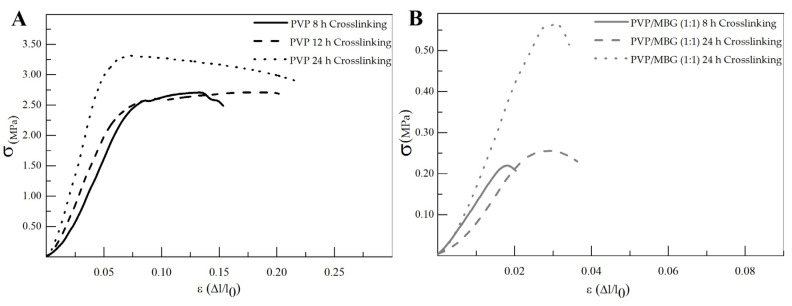
Representative stress–strain curves of plain PVP (**A**) and PVP/MBG (1:1) composite membranes (**B**) crosslinked for different times: 8, 12 and 24 h.

The different membranes of PVP and PVP/MBG (1:1) composites thermally crosslinked at 165 °C for 8, 12, and 24 h were submitted to uniaxial stress–strain tests as shown in Figure 11. These results allow us to investigate the effect of thermal crosslinking and MBG nanoparticle incorporation on the mechanical properties of the electrospun matrices. 

The comparison between plain PVP (Figure 11A) and PVP/MBG (1:1) (Figure 11B) stress–strain curves shows that mechanical properties were significantly modified with the MBG nanoparticles incorporation. With the incorporation of MBG nanoparticles, the composite membranes exhibit minimal plastic deformation as their rupture occurs almost immediately after the elastic region. This behavior is characteristic of brittle materials. The following three mechanical parameters that characterize the membranes were obtained from the stress–strain curves: Young’s modulus (E); the yield strength (σ_YS_); and the ultimate tensile strength (UTS). The results for each membrane are shown in Table 2 and corroborate the changes in the mechanical properties. The presence of MBG in the PVP fibers implies a significant reduction in the three mechanical parameters calculated. The Young’s modulus decreases by a factor of around 3 up to 3.5 times with the presence of MBG nanoparticles. This decrease along with the reduction in UTS values could be justified by the increase in the inhomogeneity in average fiber diameter, as seen in the SEM and TEM images of the composites. In fact, the poor dispersion of MBG nanoparticles at high concentrations leads to the formation of clusters, which decreases the homogeneity of PVP fibers. A higher concentration of MBG is favorable for maintaining the bioactivity of the scaffolds, but it may also contribute to the brittle mechanical properties observed in the composite samples. Reducing the amount of MBG particles inside the composite matrices leads to a trade-off between high mechanical properties and bioactivity. However, the high bioactivity of the composite fibers in necessary for BTE applications [43,44]. In fact, previous studies have indicated that there exists a certain threshold concentration of bioactive glass particles beyond which the nanoparticles can cause defects instead of reinforcing the polymer matrices, resulting in increased brittleness of the nanocomposites [40].

The time of thermal crosslinking also affected the mechanical properties of the samples. In general, the changes were very similar for plain PVP and PVP/MBG (1:1) composites. For instance, the plain PVP samples crosslinked for 8 and 12 h exhibited similar mechanical behavior under tensile forces, which also occurred for PVP/MBG (1:1) composites crosslinked for 8 and 12 h. Furthermore, when the samples were crosslinked for 24 h, the mechanical parameters reached higher values. The Young’s modulus increases in both PVP and PVP/MBG (1:1) composites by a factor of around 1.8 up to 1.9 when the crosslinking time increases from 8 to 24 h. In general, it is expected that crosslinking processes affect the mechanical properties of polymers since new interactions between polymer chains are being created. These results show that longer crosslinking time is responsible for enhancing the mechanical strength of the samples. Thus, for periods of 8 and 12 h at 165 °C, the crosslinking process is not yet complete, while 24 h of thermal crosslinking means higher bonding levels between the PVP chains.

### 3.5. Cytotoxicity Assays

**Figure 12:**
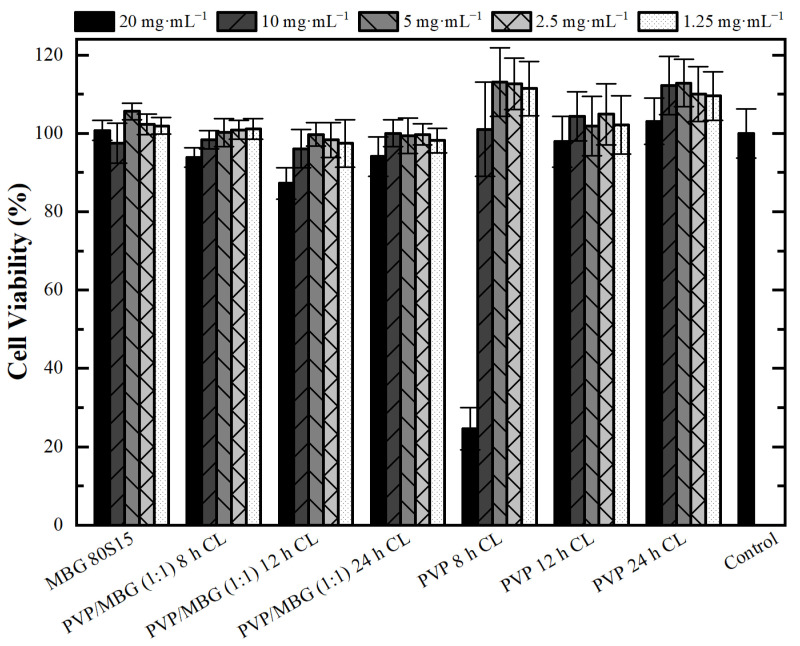
Osteoblast cell line viability after 48 h exposure to diverse types of PVP membranes through indirect method (CL stands for crosslinking). Data is expressed as average ± standard deviation for at least five independent experiments.

Cytotoxicity assays were performed to evaluate the cytotoxic effect of MBG powder, electrospun PVP and PVP/MBG (1:1) composites using the extract method. Figure 12 shows cell viability after incubation with 20 mg·mL^−1^ of each type of membrane studied and the respective two-fold dilutions. In general, the results show an absence of cytotoxic effects in Saos-2 cells, except for plain PVP membrane thermally crosslinked at 165 °C for 8 h and an extract concentration of 20 mg·mL^−1^. This may conflict with some researchers who have reported an increasing interest in PVP towards its use in biomedical applications due to its inertness, chemical stability and biocompatibility [22]. To understand these results, it is worth pointing out that PVP was previously thermally crosslinked. At elevated temperatures, the stability of PVP decreases and leads to the opening of pyrrolidone ring fractions. This phenomenon results in the transfer of hydrogen atoms from one radical to another, which leads to the formation of double bonds by hydrogen donor and saturation of acceptor by the crosslinking process [45]. Furthermore, it can be found in the literature that the biocompatibility of PVP-based materials depends on PVP content and on changes in pyrrolidone rings, which may lead to the consequent loss of biocompatibility [46]. There are few reports about the thermal crosslinking process of PVP, and the effect of MBG nanoparticle incorporation on the thermal behavior of electrospun PVP fibers. However, the results presented here may lead to the hypothesis that electrospun PVP matrices were not fully crosslinked at 165 °C for 8 h (in concordance with tensile tests), which may indicate the presence of open pyrrolidone rings, consequently decreasing its biocompatibility. However, after a two-fold dilution of the extract concentration to 10 mg·mL^−1^, cell viability rises to 100%. This is in concordance with the cell viability of the PVP/MBG (1:1) composite crosslinked in the same conditions of the above-mentioned PVP membrane (165 °C for 8 h). The concentration of 20 mg·mL^−1^ for this composite means an individual concentration equal to 10 mg·mL^−1^ of both PVP and MBG 80S15. For this concentration, both individual materials revealed no cytotoxic effects; thus the PVP/MBG (1:1) composite also revealed the absence of cytotoxicity.

### 3.6. Bioactivity Assay

The bioactivity of MBG 80S15 pellets and PVP/MBG (1:1) composites was investigated in vitro by soaking the samples in SBF to detect the formation of HA on the surface. The samples were examined by SEM, XRD and FTIR.

**Figure 13:**
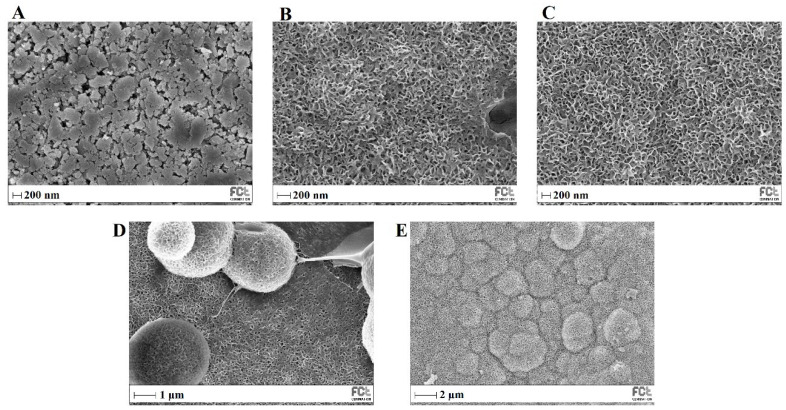
SEM images of MBG 80S15 before (**A**) and after soaking in SBF for 1 day (**B**), 3 days (**C**), 5 days (**D**) and 10 days (**E**).

Figure 13 shows SEM images of MBG 80S15 before and after different immersion intervals in SBF. Before soaking in SBF solution (Figure 13A), the MBG particle’s surface was smooth. After soaking for 24 h, the surfaces of the MBG pellets showed important changes. The surface appeared to be completely covered by a layer consisting of crystallite structures with needle-like shapes. EDS analysis confirmed that the chemical composition can be assigned to an HCA phase, given a Ca/P ratio slightly lower than 1.67 (Table 3). Furthermore, the thickness of the HCA layer continued to grow over time. After 5 days of immersion, cauliflower-like clusters with needlelike crystallites formed spontaneously to reduce surface energy. These clusters grew over time, so that after MBG soaking in SBF for 10 days the surface was completely covered in cauliflower-like clusters.

**Figure 14:**
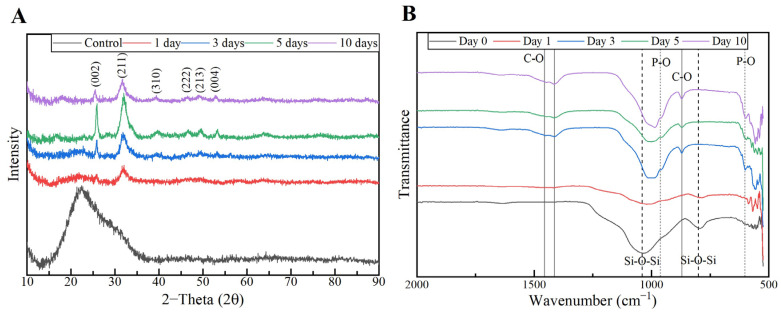
XRD patterns (**A**) and FTIR spectra (**B**) of MBG 80S15 after soaking in SBF for different periods of time.

The XRD results of the MBG 80S15 pellets soaked in SBF for 1, 3, 5 and 10 days are shown in Figure 14A. As mentioned before, MBG showed the common broad band of amorphous materials at about 15–35° (2θ) before soaking in SBF. After 1 day of immersion in SBF, the XRD pattern of MBG 80S15 showed a major peak located at 32° (2θ) and another peak located at 26° (2θ), which are assigned to the (211) and (002) reflection of an apatite-like phase [14]. After soaking for 3 days, the four new diffraction peaks at 39, 46, 49 and 53° (2θ) were assigned to the (310), (222), (213) and (004) reflections of HCA, respectively. As the immersion and mineralization time increased to 5 days, the diffraction peaks became more intense. The above-mentioned newly formed diffraction peaks are in accordance with the standard card of HCA (JCPD 24-0033). The XRD results demonstrate that MBG 80S15 has good bioactivity in vitro and its mineralization product is HCA. Furthermore, the crystallinity of HCA became higher as immersion time increased [1].

The growth of HCA on MBG 80S15 surface was further investigated by FTIR spectroscopy analysis. Figure 14B illustrates the IR spectra for MBG 80S15C samples after the same time periods soaked in SBF that were studied by SEM. Before immersion in SBF, the adsorption bands at 800 and 1040 cm^−1^ can be indexed to Si-O-Si bonds. However, when soaking in SBF for 3 days, three P–O crystalline vibrational bands near to 568, 602 and 963 cm^−1^ were detected (the noise near 500 cm^−1^ hindered the accurate analysis of the 568 cm^−1^ band). Furthermore, three other new vibrational bands were detected near to 1456, 1414 and 870 cm^−1^, which are assigned with C-O bond indicating the growth of the HCA layer on the surface of MBG 80S15 [47,48,49].

The above SEM, XRD and FTIR results indicate that the MBGs 80S15 can induce the formation of an HCA layer on their surface even for short SBF soaking periods, which corroborates the superior in vitro bone forming bioactivity, pointing out the interesting properties of the MBG 80S15 as a material for bone tissue engineering.

**Figure 15:**
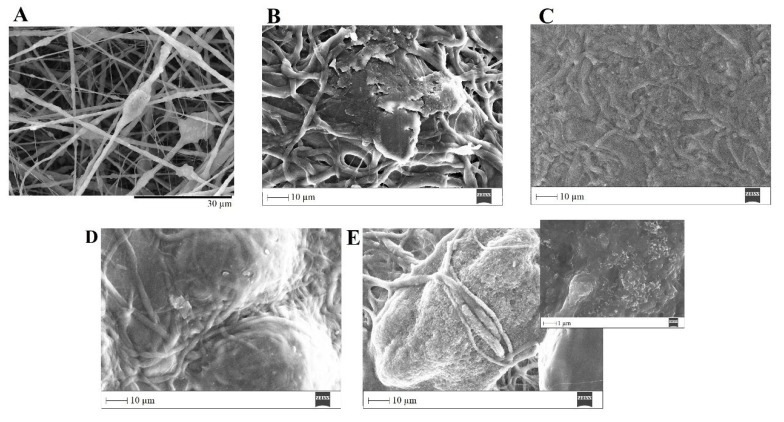
SEM images of PVP/MBG (1:1) composite crosslinked for 8 h before (**A**) and after soaking in SBF for 1 day (**B**), 3 days (**C**), 5 days (**D**) and 10 days (**E**) and respective inset micrograph at higher magnification.

**Figure 16:**
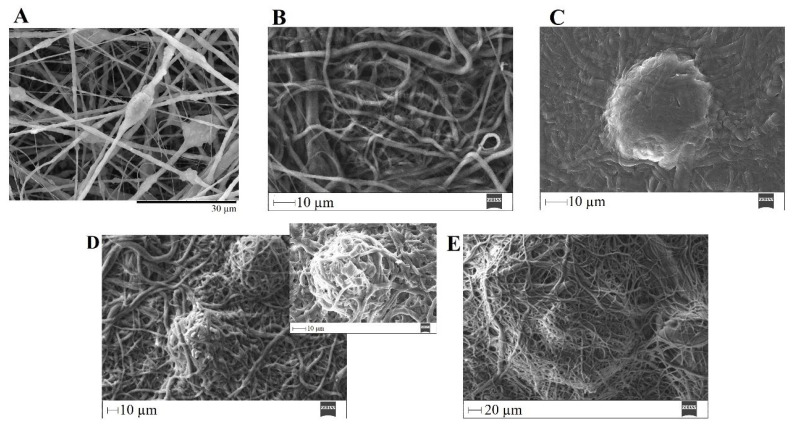
SEM images of PVP/MBG (1:1) composite crosslinked for 12 h before (**A**) and after soaking in SBF for 1 day (**B**), 3 days (**C**), 5 days (**D**) and respective inset micrograph and 10 days (**E**).

**Figure 17:**
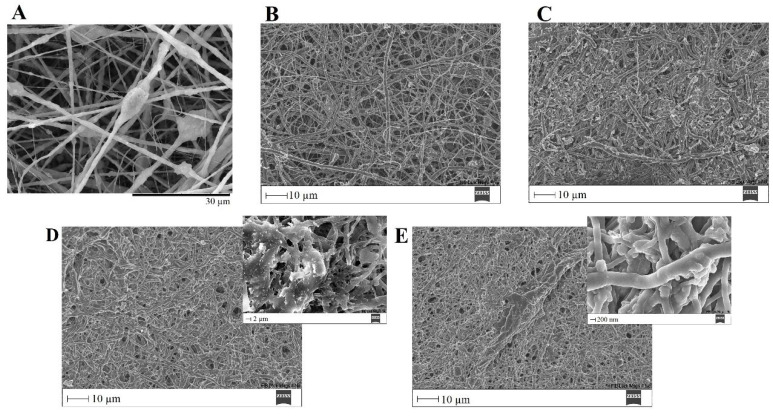
SEM images of PVP/MBG (1:1) composite crosslinked for 24 h before (**A**) and after soaking in SBF for 1 day (**B**), 3 days (**C**), 5 days (**D**) and respective inset micrograph and 10 days (**E**) and respective inset micrograph.

The ability of a biomaterial to form an HCA layer on its surface when exposed to SBF is often used to estimate its bone-bonding potential. In order to study the formation of this layer, the PVP/MBG (1:1) composite scaffolds were also analyzed by SEM, XRD and FTIR before and after being soaked in SBF. Furthermore, the bioactive behavior of the composite scaffolds is also dependent on the degradation ratio of the polymeric matrices, which in turn is dependent on the crosslinking conditions. To study the best crosslinking conditions considering the effect on enhancing the bioactivity of the scaffolds, the bioactivity assay was carried out on scaffolds crosslinked at 165 °C for 8 h (Figure 15), 12 h (Figure 16) and 24 h (Figure 17). SEM analysis reveals the surface morphology of the scaffolds before and after soaking in SBF for 1, 3, 5 and 10 days. After immersion for 1 day, the surface of the samples crosslinked for 8, 12 and 24 h becomes rougher. The samples’ porosity decreases when compared with the samples before immersion in SBF: this effect is more accentuated for the samples crosslinked for 8 and 12 h. Several precipitates were clearly observed on the nanocomposite surface over the immersion period. However, this effect is delayed in samples crosslinked for 24 h, probably due to the slower degradation of the PVP fibers hindering contact between the MBG glass particles and the medium. The bioactivity of the scaffolds can be clearly seen in the samples crosslinked for 8 and 12 h with the micrographs showing the spherical structures associated with the typical cauliflower-like structure. However, there are PVP fibers that have not completely degraded yet. Figure 15E and the respective inset micrograph show that HCA growth stretches the PVP fibers. This phenomenon can accelerate the degradation rate of the scaffolds when compared with the degradation rate studied for immersion in PBS. It is worth pointing out that the above-mentioned makes it impossible to evaluate the bioactivity of composites crosslinked for 3, 4 or 5 h since the samples did not keep their physical integrity when soaked in SBF.

EDS analysis (Table 3) of the nanofibrous mats surface before and after soaking in SBF allowed the determination of the atomic concentration of silicon, calcium and phosphorus, respectively. In general, there was a decline in silicon concentration while calcium and phosphorus concentrations increased over time for all the samples. Moreover, the earliest apatite precipitate is usually “Ca-deficient”, exhibiting a Ca/P ratio below 1.67. Throughout the HCA layer formation, the HA fraction theoretically increases, and the Ca/P ratio also increases asymptotically towards Ca/P ≈ 1.67 (for the times studied this observation is not obvious through EDS analysis) [50].

In general, the SEM analysis of the bioactivity assay of PVP/MBG (1:1) composites revealed quite similar results considering the HCA precipitation for samples crosslinked for 8 and 12 h, while the HCA precipitation on fibers’ surface seems to be delayed for the samples crosslinked for 24 h. 

**Figure 18:**
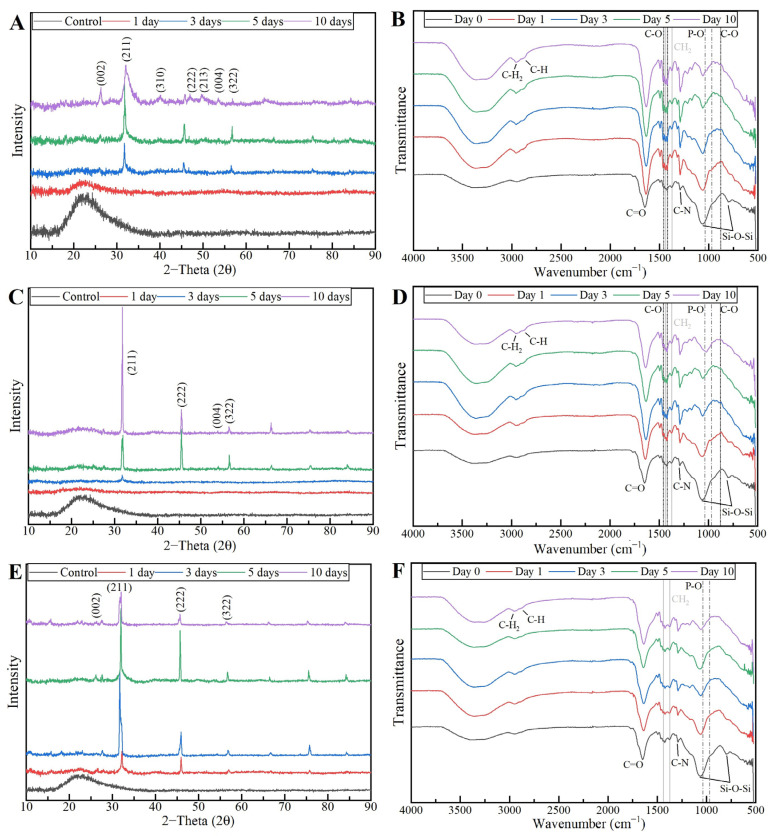
XRD patterns and FTIR spectra of PVP/MBG (1:1) composites crosslinked for 8 h (**A**,**B**), 12 h (**C**,**D**) and 24 h (**E**,**F**) after soaking in SBF for different periods of time: 1, 3, 5 and 10 days.

To confirm the formation of the HCA layer on fiber’s surface, the PVP/MBG (1:1) composites thermally crosslinked for 8, 12 and 24 h were also analyzed by XRD and FTIR before and after being soaked in SBF. XRD patterns are illustrated in Figure 18A,C,E. All samples exhibited no diffraction peaks before soaking in SBF, as expected due to the amorphous structure of both MBG and the PVP. However, significant changes in the XRD patterns occur after immersion in SBF for the three types of composite membranes. The samples crosslinked for 8 h revealed different diffraction peaks as the immersion time increased at 2θ = 26°, 32°, 40°, 46°, 49°, 53° and 57°, which correspond to the reflection of crystalline planes with the Miller indices (002), (211), (310), (222), (213), (004) and (322), respectively. In the case of composites crosslinked for 12 h, as the SBF immersion time increased, the XRD patterns show diffraction peaks at 2θ = 32°, 46°, 53° and 57°, which correspond to the reflection of crystalline planes with the Miller indices (211), (222), (004) and (322), respectively. Lastly, in the above-mentioned conditions, the XRD patterns of the composites crosslinked for 24 h show diffraction peaks at 2θ = 26°, 32°, 46° and 57°, which correspond to the reflection of crystalline planes with the Miller indices (002), (211), (222) and (322), respectively. As previously discussed for MBG 80S15 bioactivity assay, the newly formed diffraction peaks for the three types of polymeric composites studied are in concordance with the standard card of HCA (JCPD 24-0033). 

These XRD results are in accordance with FTIR analysis (Figure 18B,D,F), revealing the formation of the HCA structure. Starting with the FTIR spectrum of PVP/MBG (1:1) before immersion in SBF, the following absorption bands are assigned to PVP: the band located around 1648 cm^−1^ can be ascribed to the stretching vibration of the C=O in the pyrrolidone group; the broad band near to 2850–3000 cm^−1^ is associated with CH stretching modes, which can be assigned to five overlapping signals: asymmetric CH_2_ stretching (polymer chain: 2983 cm^−1^, ring: 2954 cm^−1^), symmetric CH_2_ stretching (polymer chain: 2919 cm^−1^; ring: 2885 cm^−1^) and ternary CH (2852 cm^−1^); the bands at 1427 cm^−1^ and 1372 cm^−1^ also correspond to the CH deformation modes from the CH_2_ group; the absorption bands at 1288 cm^−1^ are related to C–N bending vibration from the pyrrolidone structure [51]. The absorption bands at 470 (not seen in the graphs due to noise), 802 and 1058 cm^−1^ can be assigned to Si-O-Si bonds of the MBG 80S15. After immersion in SBF, the membranes crosslinked for 8 and 12 h showed similar FTIR spectra over time: the P–O symmetric stretching vibrational bands near 970 cm^−1^ were detected (the noise near 500 cm^−1^ hindered the accurate analysis of the other two characteristics bands of the P–O bond: 568 and 600 cm^−1^, associated with bending modes); after 5 days of immersion in SBF, the vibrational band near 970 cm^−1^ was no longer detected due to the shift of the 1058 cm^−1^ band assigned to Si-O-Si band. This shift was related to the overlap of a new band at 1036 cm^−1^, which is associated with the asymmetric stretching of P–O bond [52]; three vibrational bands near 1458, 1417 and 880 cm^−1^ were also detected and assigned to the C-O bond. However, it was noticed that the bands near 1458 and 1417 cm^−1^ increased in intensity over immersion time, while the band at 880 cm^−1^ revealed only a slight change in intensity.

The above-mentioned changes in the FTIR spectra were not detected for the samples crosslinked for 24 h, except for the shift associated with the new band at 1036 cm^−1^ after 10 days after immersion in SBF. The FTIR analyses are in accordance with the SEM results, indicating the growth of the HCA layer over time, which seems to be delayed for the scaffolds crosslinked for 24 h.

Hench has reported a detailed analysis of the reactions involved in the five-stage mechanism of apatite formation upon contact of BGs with SBF. The five stages include: (1) rapid ion exchange of alkali ions with hydrogen ions from the liquid medium, (2) dissolution of the glass network, (3) polymerization of silica gel and (4 and 5) chemisorption and crystallization of the carbonated hydroxyapatite layer [53]. Stages (1) and (2) explain the fading of the band at 802 cm^−1^ (Si-O-Si), which was noticed in all the scaffolds studied. Furthermore, stages 1 and 2 strongly depend on PVP degradation rate, thus allowing us to state that crosslinking directly affects the bioactivity of the polymeric composite scaffolds studied. On the other hand, as shown in the swelling assay, the high wettability of PVP may favor SBF interaction with MBG nanoparticles, allowing apatite mineralization inside PVP fibers and subsequent growth in the radial direction [54].

The findings of this study are consistent with previous research that has demonstrated that the inclusion of BG particles in polymeric matrices enhances the in vitro formation of hydroxyapatite on the surface of nanocomposites when exposed to an SBF medium. For instance, Lin et al., [55], Allo et al., [56] and Liverani et al. [43] performed different studies and achieved similar conclusions. The researchers found that the incorporation of bioactive glasses into PCL [55,56] and PCL/chitosan [43] nanofibrous matrices significantly enhanced their apatite-formation ability in SBF compared with individual polymeric membranes. In addition, according to Han et al. [57], composite nanofibers demonstrated superior bioactivity compared to pure PAN-based carbon nanofibers. Additionally, Yang et al. [58] observed that the addition of BG nanoparticles to carbon nanofiber composites increased the rate of heterogeneous apatite nucleation [54].

## 4. Discussion and Conclusions

In general, the main goals proposed here have been achieved, confirming the hypothesis put forward at the beginning of this study. The composites produced can be used as bone scaffolds considering their inherent properties.

The characterization of the MBG 80S15 produced revealed the desired properties for BTE, i.e., non-toxic effects on the Saos-2 cell line and enhanced bioactivity considering the rapid HCA layer formation within the first 24 h after immersion in SBF. This is directly related to the high surface area of the MBG 80S15 powder due to its mesoporosity, thereby increasing MBG reactivity through the accelerated ion exchange with the medium.

The MBG 80S15 powder was successfully incorporated into the PVP-fibrous mats by means of electrospinning. The resulting composite materials were thermally crosslinked to reduce PVP water solubility, aiming to tailor the degradation ratio of PVP and PVP/MBG (1:1) mats. The literature reports the formation of some radicals in the temperature range of 150 to 200 °C, which can be attributed to the dissociation of methylene of the pyrrole ring or the methine of the main polymer chain. As a result, crosslinks in the polymer chains are formed [59,60].

In general, all PVP/MBG (1:1) composites thermally crosslinked at 165 °C for 8, 12 and 24 h showed HCA-forming ability. However, the impact of thermal crosslinking duration on scaffolds’ bioactivity should be considered as previously discussed. When the scaffolds were thermally crosslinked at 165 °C for 8 and 12 h, the bioactivity displayed similar results, whereas the scaffolds crosslinked for 24 h showed slower reactivity. Future experiments should be carried out to study the relation between thermal crosslinking conditions and ion release from the composites to the medium, in order to compare the HCA layer formation.

These polymeric scaffolds also revealed the absence of cytotoxic effects on Saos-2 cells as intended for BTE applications. Moreover, the degradation ratio and swelling capacity of the resulting composites can be tailored by varying the amount of MBG powder incorporated or the crosslinking conditions. It is worth pointing out the importance of thermal crosslinking conditions due to the direct relationship between swelling capacity, degradation ratio and MBGs’ ion exchange with the medium. Consequently, the bioactivity of the composites depends on crosslinking conditions.

The overall results indicated that the described electrospun nanocomposite fiber mats are promising bone scaffolds, as they combine the high bioactivity of MBG 80S15 nanosized powder and the benefit of the flexibility of electrospun PVP mats. The major hurdle to face is to combine bioactivity and mechanical strength, since the results showed a trade-off between bioactivity and mechanical strength in PVP/MBG (1:1) scaffolds. This effect resulted from the inhomogeneity of PVP nanofibers’ diameter with the incorporation of large amounts of MBG powder, which tends to agglomerate and form clusters.

For future work, it would be interesting to study the properties of these composites using in vivo models, in order to assess the ability of the scaffolds to induce osteogenesis and angiogenesis. Moreover, it would be of great interest to encapsulate other biomaterials within PVP fibers, such as magnetic nanoparticles or drugs, in order to perform local treatment of bone disease through magnetic hyperthermia or drug delivery and simultaneously induce bone regeneration.

Considering the results presented, the scaffolds thermally crosslinked for 12 h showed the most promising results. These scaffolds showed rapid HCA layer formation, which was comparable to that obtained for scaffolds thermally crosslinked for 8 h. However, the composites crosslinked for 12 h are easier to handle, e.g., they keep their physical properties for longer periods when immersed in SBF solution. They also revealed high biocompatibility and suitable swelling capacity for further drug delivery applications combined with BTE.

## Figures and Tables

**Table 1:** Range of four independent variables in a custom design of experiments (JMP software, version 13.0) aiming to minimize average electrospun PVP fiber diameter and respective desirable response using RSM.

Variable	Type	Range	Desirable Response	Estimated Output (nm)
Applied voltage (kV)	Continuous	15–20	18.6	652 ± 179
Flow rate (mL·h^−1^)	Continuous	0.1–0.4	0.1	
Tip-collector distance (cm)	Continuous	15–20	17.3	
Needle gauge	Categorical	G23; G27	G27	

**Table 2:** Calculated mechanical parameters for each membrane type: Young’s modulus (E), ultimate tensile strength (UTS) and yield strength (σ_Y_).

Sample	E (MPa)	UTS (MPa)	σ_Y_ (MPa)
PVP 8 h crosslinking	39.2 ± 10.9	2.45 ± 0.50	2.77 ± 056
PVP 12 h crosslinking	45.7 ± 14.1	2.27 ± 0.35	2.75 ± 0.44
PVP 24 h crosslinking	70.0 ± 16.2	3.01 ± 0.28	3.39 ± 0.38
PVP/MBG (1:1) 8 h crosslinking	11.9 ± 3.4	0.22 ± 0.07	0.23 ± 0.05
PVP/MBG (1:1) 12 h crosslinking	13.2 ± 2.5	0.23 ± 0.03	0.26 ± 0.05
PVP/MBG (1:1) 24 h crosslinking	22.9 ± 3.9	0.58 ± 0.09	0.59 ± 0.07

**Table 3:** Atomic concentration (At. %) of silicon (Si) and respective Ca/P ratio during the bioactivity assay of the different types of membrane studied and MBG 80S15 pellets before (control) and after soaking in SBF for 1, 3, 5 and 10 days. These results were obtained from EDS analysis (CL stands for crosslinking).

EDS (At. %)	Control		1 Day		3 Days		5 Days		10 Days	
Sample	Ca/P	Si	Ca/P	Si	Ca/P	Si	Ca/P	Si	Ca/P	Si
MBG 80S15 pellet	1.16	75.39	1.60	69.76	1.59	9.01	1.52	8.46	1.58	2.24
PVP/MBG (1:1) 8 h CL	1.49	73.59	1.48	89.89	0.75	85.96	1.23	34.48	1.64	0.60
PVP/MBG (1:1) 12 h CL	1.49	73.59	1.05	92.32	1.13	36.11	1.58	31.73	1.83	5.75
PVP/MBG (1:1) 24 h CL	1.49	73.59	1.43	85.69	1.47	77.99	3.72	85.66	1.22	66.24

## Data Availability

The data presented in this study are available on request from the corresponding author.
